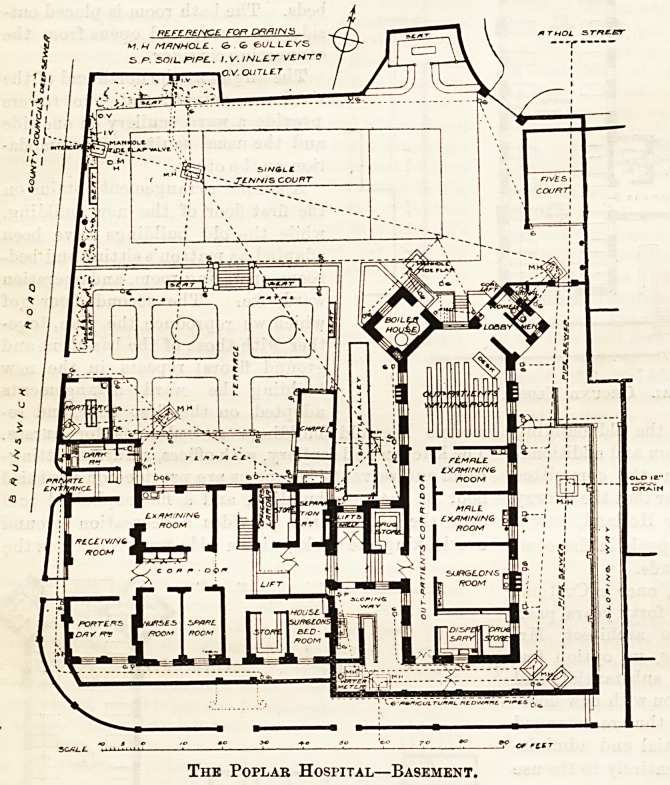# The Poplar Hospital for Accidents

**Published:** 1895-04-27

**Authors:** 


### The Institutional Workshop.

### HOSPITAL CONSTRUCTION.

### THE POPLAR HOSPITAL FOR ACCIDENTS.

This institution has been reconstructed in accordance with the plans which accompany this notice, partly on additional ground adjoining the site of the

The Poplar Hospital - Ground Floor.

old building, partly by remodelling the old building itself. The necessity for reconstruction and additional accommodation has forced itself on the committee since 1890, but it was not till last year that the energy of their chairman, the Hon. Sydney Holland, saw a successful issue to his labours and appeals in the completion of the building as it now stands.

The condition of the old structure, once a Customs House, then a hotel, and then, for forty years past, adapted to hospital work, left the architect, Mr. Rowland Plumbe, of Fitzroy Square, no option but to gut the interior, make the walls substantial, and re-arrange the internal accommodation with new flooring and staircases. The old building thus re-arranged is almost entirely devoted to residential and administrative work, and the new buildings entirely to the use of patients.

The ground floor of the old building is raised some feet above the street, and the site itself, falling from south to north, gives level access to the receiving room for accidents, with examining room adjoining, in the basement of the old structure. A lift, close at hand, affords communication for "cases" to the patients' wards on the upper floors.

The remainder of the basement of the old building is divided into rooms for hospital officials and a separation ward, a corridor, with steps to meet the difference in levels, giving access to the basement floor of the new building. This is entirely devoted to the out-patient department, which is thus kept entirely distinct from the rest of the hospital, and is provided with distinct exits and entrances for out-patients by sloping ways to the street. The department comprises a waiting room, male and female examining rooms, a surgeons' room, and a small dispensary and drug store.

On the ground floor of the old building, accommodation is provided for the secretary and for a matron's office, a surgeon's sitting room (furnished with a billiard table), dining-room, library, &c. One of the old wards is kept as a separation ward, and a connecting corridor, lighted on one side, leads to one of the new wards for twenty beds. The bath room is placed outside the ward, and opens from the connecting corridor.

The angles at the north end of the ward are cut off, and two towers provide a ward scullery on one side and the usual sanitary accommodation on the other.

A similar arrangement obtains on the first floor of the new building, while the old buildings have been adapted as matron's sitting and bedroom, operating room, and operation ward, &c. The second floor (of which we reproduce the plan, together with those of the basement and ground floors) repeats in the new building the ward arrangements adopted on the first floor, and remodels the old buildings as bedrooms for nurses. The kitchen and scullery, with offices, a nurses' sitting-room and servants' bedrooms are provided on the third floor of the new building, and a flat asphalted roof over the old portion provides a recreation ground overlooking the docks, with a wide prospect across the

The Poplar Hospital—Second Floor.

river to Greenwich and Woolwich, and to the Kent and Surrey hills.

An iron escape staircase stands between the towers already mentioned at the northern end of the wards, and an external lift from the courtyard delivers goods to the kitchens and conveys dust and ward refuse from each floor to the furnaces in the boiler-rooms in the basement. A pair of lifts, centrally placed in the connecting corridor, supply a communication between the kitchen and every floor of the buildings, both new and old.

The whole of the arrangements exhibit sound sense and considerable skill on the part of the architect, and are generally in accordance with the latest developments of hospital planning. We cannot help regretting that the position of the bath-room on each floor, which is unusual but has good points in view of the special purpose of this hospital, prevents the completion of the recognised system of fenestration in relation to the beds in the wards, and we may add

The Poplar Hospital—Basement.

that a dressing-room or rooms in connection with the examining rooms of the out-patients' department would seem desirable. The dispensary appears small, and is obviously not in the best place for hospital use as well as outside dispensing, but probably the fact that the hospital is for accidents *only* explains both objections. It would be out of place to criticise the operating-room, and the position of some of the sanitary appliances in the old buildings; these are but temporary arrangements, as we understand that an operating theatre, a post-mortem room, and separation block, with its nurses' rooms and other buildings have yet to be built. The fact that the building as it now stands is free from debt is a matter of most sincere congratulation, and, we venture to think, of rarer occurrence than ought to be the case with such institutions.

## Figures and Tables

**Figure f1:**
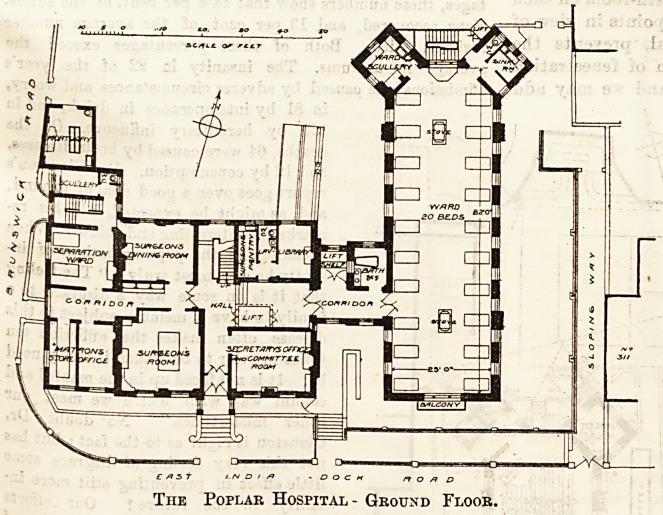


**Figure f2:**
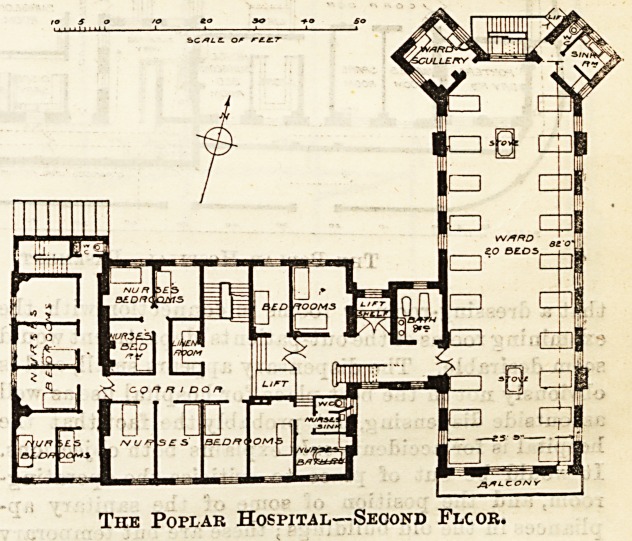


**Figure f3:**